# IQGAP1 Is Involved in Post-Ischemic Neovascularization by Regulating Angiogenesis and Macrophage Infiltration

**DOI:** 10.1371/journal.pone.0013440

**Published:** 2010-10-15

**Authors:** Norifumi Urao, Masooma Razvi, Jin Oshikawa, Ronald D. McKinney, Rupal Chavda, Wadie F. Bahou, Tohru Fukai, Masuko Ushio-Fukai

**Affiliations:** 1 Department of Pharmacology, Center for Lung and Vascular Biology, Center for Cardiovascular Research, University of Illinois at Chicago, Chicago, Illinois, United States of America; 2 Departments of Medicine and Pharmacology, Center for Cardiovascular Research, University of Illinois at Chicago, Chicago, Illinois, United States of America; 3 Department of Medicine, State University of New York at Stony Brook, Stony Brook, New York, United States of America; Leiden University Medical Center, Netherlands

## Abstract

**Background:**

Neovascularization is an important repair mechanism in response to ischemic injury and is dependent on inflammation, angiogenesis and reactive oxygen species (ROS). IQGAP1, an actin-binding scaffold protein, is a key regulator for actin cytoskeleton and motility. We previously demonstrated that IQGAP1 mediates vascular endothelial growth factor (VEGF)-induced ROS production and migration of cultured endothelial cells (ECs); however, its role in post-ischemic neovascularization is unknown.

**Methodology/Principal Findings:**

Ischemia was induced by left femoral artery ligation, which resulted in increased IQGAP1 expression in Mac3^+^ macrophages and CD31^+^ capillary-like ECs in ischemic legs. Mice lacking IQGAP1 exhibited a significant reduction in the post-ischemic neovascularization as evaluated by laser Doppler blood flow, capillary density and α-actin positive arterioles. Furthermore, IQGAP1^−/−^ mice showed a decrease in macrophage infiltration and ROS production in ischemic muscles, leading to impaired muscle regeneration and increased necrosis and fibrosis. The numbers of bone marrow (BM)-derived cells in the peripheral blood were not affected in these knockout mice. BM transplantation revealed that IQGAP1 expressed in both BM-derived cells and tissue resident cells, such as ECs, is required for post-ischemic neovascularization. Moreover, thioglycollate-induced peritoneal macrophage recruitment and ROS production were inhibited in IQGAP1^−/−^ mice. *In vitro*, IQGAP1^−/−^ BM-derived macrophages showed inhibition of migration and adhesion capacity, which may explain the defective macrophage recruitment into the ischemic tissue in IQGAP1^−/−^ mice.

**Conclusions/Significance:**

IQGAP1 plays a key role in post-ischemic neovascularization by regulating, not only, ECs-mediated angiogenesis but also macrophage infiltration as well as ROS production. Thus, IQGAP1 is a potential therapeutic target for inflammation- and angiogenesis-dependent ischemic cardiovascular diseases.

## Introduction

Neovascularization in response to ischemia is an important repair process, which is dependent on inflammation and angiogenesis, defined as the process of new vessel formation from pre-existing capillary-like endothelial cells (ECs) as well as arteriogenesis [1]. Macrophage infiltration into ischemic tissues plays a key role in ischemia-induced neovascularization by releasing angiogenic cytokines including vascular endothelial growth factor (VEGF) [1], [2], [3], [4]. They also protect tissues from necrosis, and promote healing/regeneration of muscles during limb ischemia [3], [5], [6], [7]. VEGF induces angiogenesis by stimulating EC migration and proliferation primarily through the VEGF type2 receptor (VEGFR2) [8], as well as by upregulating stromal derived factor-1α (SDF-1α) in ischemic sites, thereby recruiting proangiogenic myeloid cells [9]. Impaired migratory response of bone marrow (BM)-derived cells contributes to inhibition of neovascularization capacity of those cells [10]. We previously reported that reactive oxygen species (ROS) derived from NADPH oxidase expressed in inflammatory cells and ECs play a critical role in ischemia-induced neovascularization [11]. Thus, identifying critical regulators for macrophage-mediated inflammatory responses and angiogenesis is important for understanding the mechanisms of post-ischemic neovascularization and treatment of ischemic heart and limb diseases.

IQ-domain GTPase-activating protein 1 (IQGAP1) is a scaffold protein that plays a pivotal role in regulating actin cytoskeleton and cell migration by interacting directly with actin, active Rac1/Cdc42, Arp2/3 and N-WASP [12], [13], [14]. In actively migrating cells, IQGAP1 accumulates at the leading edge and cross-links actin filaments [15], [16]. IQGAP1 also binds to actin filament nucleator, Diaphanous-related formin (Dia1), and thus localizing Dia1 at the actin-rich phagocytic cup, which is required for migration and phagocytosis of RAW macrophage [17]. We previously demonstrated that IQGAP1 directly binds to VEGFR2 [18] and it plays a critical role in transmitting VEGF-mediated signals to the angiogenesis-related responses in ECs [18], [19]. We have shown that IQGAP1 translocates to the leading edge where it binds to VEGFR2 and NADPH oxidase2, a major source of ROS in ECs, thereby promoting ROS production and directional EC migration [18], [20]. Moreover, we found that IQGAP1 expression is dramatically increased in the regenerating ECs layers after vascular injury [18] as well as in capillary-like ECs during angiogenesis after hindlimb ischemia [21]. Meyer et al. recently showed that knockdown of IQGAP1 expression with siRNA inhibits VEGF-induced angiogenesis in an *in vivo* model of chicken chorioallntoic membrane [19]. IQGAP1 has been implicated in cancer and tumorigenesis [22]. However, the role of IQGAP1 in post-ischemic neovascularization remains unknown.

In the present study, we demonstrate that IQGAP1 expression is increased in infiltrated macrophages and ECs in ischemic tissues. Mice lacking IQGAP1 show reduced limb blood flow recovery, capillary density and α-actin positive arterioles, as well as tissue repair. These are due to decreased macrophage infiltration and ROS production in ischemic muscles in IQGAP1^−/−^ mice. Moreover, thioglycollate-induced peritoneal leukocyte recruitment and its ROS production are significantly inhibited in IQGAP1^−/−^ mice. BM reconstitution experiments show that IQGAP1 in both inflammatory cells and ECs are necessary to increase blood flow recovery, capillary density and α-actin positive vessels. *In vitro*, IQGAP1^−/−^ BM-derived macrophages show inhibition of migration due to impaired actin polarization as well as adhesion capacity to fibronectin and vitronectin. Thus, endogenous IQGAP1 plays an important role in ischemia-induced revascularization by regulating macrophage infiltration and EC-mediated angiogenesis.

## Methods

### Ethics Statement for Animal Study

Study protocols were approved by the Animal Care and Institutional Biosafety Committee of University of Illinois at Chicago (ACC: 09-066).

### Animals

IQGAP1^−/−^ mice on mix background [23] were backcrossed with C57BL/6 mice 10 generations. Age matched C57BL6 mice used for control, wild type (WT) mice, were purchased from Jackson Laboratory. All mice were maintained at the University of Illinois at Chicago animal facilities. Mice at 8 to 12 weeks old were used for experiments.

### Hindlimb ischemia model

Mice were subjected to unilateral hindlimb surgery under anesthesia with intraperitoneal administration of ketamine (100 mg/kg) and xylazine (10 mg/kg). We performed ligation and segmental resection of left femoral vessels followed by physiological and histological analysis as described [24]. Briefly, the left femoral artery was exposed, ligated both proximally and distally using 6-0 silk sutures and the vessels between the ligatures was excised without damaging the femoral nerve. All arterial branches between the ligations were obliterated using an electrical coagulator (Fine Scientific Tools). Skin closure was done using 6-0 nylon sutures. We measured ischemic (left)/nonischemic (right) limb blood flow ratio using a laser Doppler blood flow (LDBF) analyzer (PeriScan PIM 3 System; Perimed) as we described [25]. Mice were anesthetized and placed on a heating plate at 37°C for 10 minutes to minimize temperature variation. Before and after surgery, LDBF analysis was performed in the plantar sole. Blood flow was displayed as changes in the laser frequency, represented by different color pixels, and mean LDBF values were expressed as the ratio of ischemic to nonischemic LDBF. The incidence and severity of hind limb tissue necrosis was assessed after surgery at indicated time point, severity of necrosis was assessed using the following scale: 0 =  no necrosis, 1 =  one toe, 2 =  two or more toes, 3 =  foot necrosis, 4 =  leg necrosis, and 5 =  autoamputation of the entire leg as described [26].

### Histological analysis

For cryosections, mice were euthanized and perfused through the left ventricle with saline and 4% paraformaldehyde, limbs were fixed in 4% paraformaldehyde (PFA) overnight and incubated with 30% sucrose, and adductor and gastrocunemius muscles were embedded in OCT compound (Sakura Finetek). For paraffin sections, we performed methanol fixation or PFA fixation with decalcification by Immunocal (Decal Chemical Corp.). Capillary density in the ischemic muscles was determined in 5 µm cryosections or methanol fixed paraffin sections that were stained with anti-mouse CD31 antibody (BD). Arterioles were stained with Cy3-conjugated anti-αSMA antibody (1A4, Sigma), or anti-αSMA antibody (ASM-1, American Research Products) followed by biotinylated anti-mouse IgG antibody (Vector Laboratories). Monocytes/macrophages were labeled with anti-Mac3 antibody followed by biotinylated anti-rat IgG (Vector Laboratories). For immunohistochemistry, we used R.T.U. Vectorstain Elite (Vector Laboratories) followed by DAB visualization (Vector Laboratories). Images were captured by Axio scope microscope (Zeiss) or confocal microscopy (Zeiss) and processed by AxioVision 4.8 or LSM510 software (Zeiss), respectively.

### Thioglycollate-induced peritonitis

Mice were intraperitoneally injected with 1 ml of 4% thioglycollate broth (Sigma). Four days after the injection, mice were killed by CO_2_ inhalation. Cells in peritoneal cavity were recovered by peritoneal lavage by injecting intraperitoneally with 3 changes of 3 ml of PBS containing 0.1% BSA, 0.5 mM EDTA and 10 U/ml of heparin as described previously [27]. Total cell and macrophage count were determined by Neubauer hemocytometer under microscopy.

### Lucigenin-based O_2_^•−^ measurement

Lucigenin-enhanced chemiluminescence assay was performed to measure O_2_^•−^ production, as we described [11], [25]. Freshly excised adductor muscles or harvested peritoneal cells (1 million cells) were placed in scintillation vials containing Krebs-HEPES buffer with 5 µM lucigenin. Light emission was detected with a scintillation counter programmed in out-of-coincidence mode. The mean chemiluminescence yields observed during a period of 30 minutes after addition of the samples were used to estimate rates of production of O2 ^•−^.

### O_2_^•−^ detection in mice

Dihydroethidium (Invitrogen) was prepared as a 1 mg/ml solution in 1% DMSO and administered at 1 mg per kg of body weight by intraperitoneal injection as reported previously[28]. Mice were killed and perfusion-fixed with 4% paraformaldehyde 30 minutes after DHE injections. Frozen sections of ischemic muscles were prepared and observed with confocal microscope (Zeiss) with excitation at 510–550 nm and emission >585 dsx7Anm to detect oxidized ethidium.

### Isolation and primary culture of bone marrow (BM)-derived macrophages

Bone marrow (BM) mononuclear cells (BMCs) were harvested from tibiae and femurs of 8 to 12 week-old IQGAP1^−/−^ and WT mice as we described previously [25]. Briefly, the BMCs were flushed from the bone with a 27G needle connected to a syringe filled with DPBS with penicillin/streptomycin and 2% FBS. Following density gradient separation by Histopaque 1077 (Sigma), the cells were cultured in DMEM medium (Gibco) supplemented with antibiotics, 10% FBS and 10 ng/ml M-CSF-1 (Peprotech). Non-adherent cells were collected after 24 hours, seeded on coverslips and differentiated for 7 days in polystyrene culture plates. The resulting BM-derived macrophages (BMMs) population was determined by staining with anti-CD11b (BD) and anti-F4/80 (BioLegend) as assessed by flow cytometry (DAKO) as described previously [29], [30].

*Western blotting*- Western blotting was performed as described previously [31]. Briefly, excised adductor muscles and isolated BMCs obtained from IQGAP1^−/−^ and WT mice at 0, 3 and 7 days after hindlimb ischemia were homogenized, and lysates were used for Western blotting analysis with primary antibodies.

### BM transplantation

BMCs were isolated by density gradient separation. Recipient mice were lethally irradiated with 9.5 Gy and received an intravenous injection of 3 million donor bone marrow cells 24 hours after irradiation. To determine the transplantation efficiency, transplantation was performed between CD45.1 mice (Jackson Laboratories) and ether IQGAP1 KO or WT mice and peripheral white blood cells were stained with anti-CD45.1 (BD) and anti-CD45.2 (BD). Hindlimb ischemia was induced 6 to 8 weeks after bone marrow transplantation.

### Cell migration assays

Migration assays were performed in a modified Boyden chamber, as described previously [25]. Briefly, BMMs were starved in M-CSF-1 overnight and serum for 4 hours before detaching and collecting by scraping. The lower chamber was filled with SDF-1α (PeproTech, 100 µg/ml) in RPMI, the upper chamber with monocytes/macrophage (0.5×10^6^/ml). Cells migrated for 4 hours in the presence or absence of SDF-1α. Cells were quantified under the microscope by blinded investigators. Two separate experiments were performed.

### Immunostaining in BMMs

BMMs were obtained as described above. After serum and M-CSF-1 starvation at 37°C for 4 hour, SDF-1α (100 ng/ml) or PBS were added and incubated for 2 minutes. After fixation with 2% paraformaldehyde, permeablization by 0.05% tritonX-100 solution and blocking in 3% BSA for 1 hour at room temperature, cells were stained by Alexa-568 conjugated anti-phalloidin (Invitrogen) and anti-IQGAP1 (Santa Cruz). For IQGAP1, primary antibody was labeled with TRITC conjugated anti-rabbit IgG (Jackson Immuno Research). Images were taken by confocal microscopy (Zeiss).

### Flow cytometory (FACS) analysis

Peripheral blood was collected in heparinized hematocrit capillary tubes (Fisher scientific) and placed in 150 µl 2% Dextran T500 in PBS plus 150 µl 3 mg/ml EDTA in PBS with 1 unit/ml heparin. The peripheral blood was allowed to settle for 30 min at room temperature. Then the top phase was collected and spun down at 500 *g* for 5 min at 4°C. The supernatant was discarded and the cells were resuspended in 1 ml of RBC lysis buffer (9 ml 0.16 M NH_4_Cl +1 ml 0.17 M Tris-Cl, pH 7.65) for 10 min at room temperature. Samples were then washed with DPBS/2% FCS and centrifuged at 500 *g* for 5 min at 4°C. Cells were resuspended in cold DPBS/2% FCS and placed on ice. Total leukocytes in the peritoneal cavity were collected as described above. Cells were stained with the appropriate antibodies; Ly-6C, Ly-6G, Flk-1, CXCR4, Sca-1 and CD11b (BD Pharmingen); cKit, Gr-1 and F4/80 (eBioscience); VEGFR1 (R&D systems). The cells were then washed, resuspended in 2 µg/ml propidium iodide in DPBS/2% FCS, and analyzed on a flow cytometer (DAKO ADP Cyan). Summit (DAKO) or FlowJo 7.6 software was used for population analysis.

### Isolation of monocytes/macrophages from muscle

Excised tibiaris anterior muscles were dissociated in DPBS containing collagenase B 0.2%(w/v) (Roche Diagnostics) and 20 mM HEPES at 37°C for 1 hour followed by passing through the 18G and the 23G needles, filtered and counted. The cells suspension was stained with specific antibodies and analyzed on a flow cytometer described above.

### Quantitative RT-PCR

Total RNA was prepared from cells or tissues using Tri Reagent (Molecular Research Center Inc.). Reverse transcription was carried out using high capacity cDNA reverse transcription kit (Applied biosystems). Quantitative PCR was performed with the ABI Prism 7000, the SYBR Green PCR kt (Qiagen) and the QuantiTect Primer Assay (Quiagen) for specific genes. Expression of genes was normalized and expressed as fold-changes relative to GAPDH.

### Cell-matrix Adhesion Assay

Ninety-six-well plates were coated over night at 4°C with 5 µg/ml purified human fibronectin (Sigma), 5 µg/ml human vitronectin (Chemicon International), 10 µg/ml human type IV collagen (Chemicon International), 10 µg/ml human fibrinogen (Enzyme Research Laboratories) or 10 µg/ml soluble recombinant human ICAM-1 (Axxora Platform), and then blocked for 1 hour at room temperature with 0.5% (w/v) heat-inactivated bovine serum albumin (BSA) in PBS. Cultured BMMs were detached from the culture plates with 1 mM EDTA in PBS after serum and M-CSF-1 starvation at 37°C for 4 hour. Cells suspended with PBS (with Ca^++^ and Mg^++^) were seeded at 2.5×10^5^ cells/well in 100 µl in the coated wells for 30 min. Non-adherent cells were removed by washing, and adherent cells were stained by 0.05% crystal violet and quantified in duplicates by light absorbance (562 nm).

### Statistical analysis

All the experiments were repeated at least three times, and all values were expressed as means. Blood flow recovery in the ischemic hindlimb was compared between the two groups by two-way repeated measures ANOVA, followed by Bonferroni post hoc analysis. Comparison between groups was analyzed by unpaired Student 2-tailed t test (2 groups) or ANOVA for experiments with more than 2 subgroups followed by Bonferroni post hoc analysis. P<0.05 was considered as statistically significant.

## Results

### IQGAP1 is involved in Neovascularization in Response to Hindlimb Ischemia

**Figure 1:**
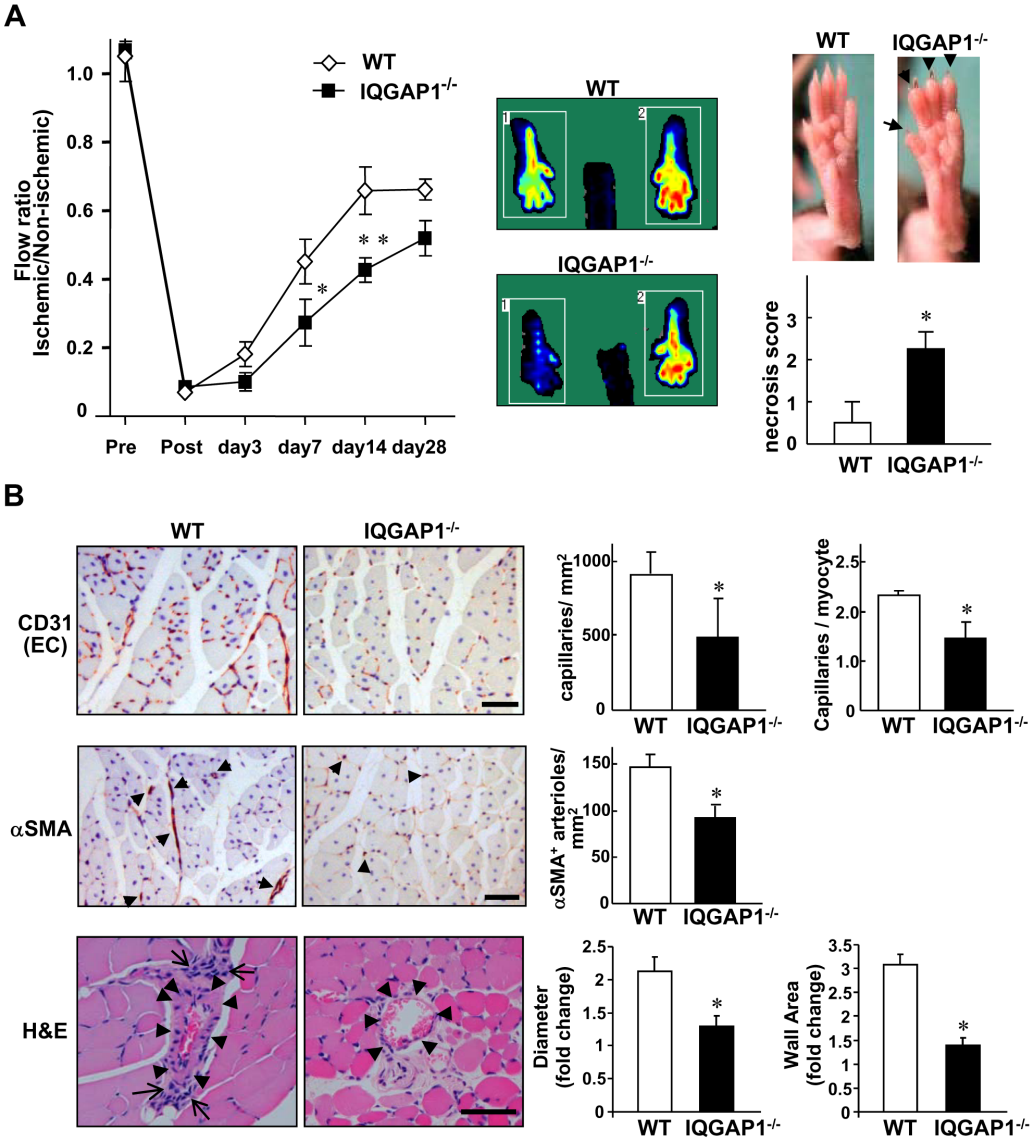
Post-ischemic revascularization is impaired in IQGAP1^−/−^ mice. **A**, Left, blood flow recovery after hindlimb ischemia in wild-type (WT) and IQGAP1^−/−^ mice, as determined by relative values of laser Doppler perfusion of the plantar sole between ischemic (left) and non-ischemic (right) legs (WT, n = 13; IQGAP1^−/−^, n = 10, **p<0.01 and *p<0.05). Middle panels show representative perfusion images at day 14. Right, tissue necrotic score on the ischemic feet was determined (n = 10 *p<0.05). In IQGAP1^−/−^, the necrotic score represents severe phenotype with edematous fingers and degenerative nail beds (black arrows). **B**, Tissues were harvested at day 14 for histological analysis. CD31 positive capillary-like ECs (top) and α-smooth muscle actin (αSMA) positive arterioles (middle) in ischemic gastrocunemius muscles. Positive staining (red brown) and nuclei (purple) are shown. Bars represent 50 µm. The graphs represent the number per mm in 2 area and/or per muscle fibers (n = 4, *p<0.05). Hematoxyline and eosin (H&E) staining of adductor muscles (bottom). Arrowheads show collateral vessels in semimembranosus muscle. Prominent cellular infiltration in adventitia and perivascular area in WT mice are shown by arrows (left). The diameter and wall area are calculated from the measurements of luminal and perivascular tracing (right). Data shown are mean+/−SEM.

To examine the role of endogenous IQGAP1 in post-ischemic neovascularization, wild-type (WT) and IQGAP1^−/−^ mice were subjected to unilateral hindlimb ischemia. Laser Doppler blood flow (LDBF) analysis showed that the blood flow recovery in ischemic feet at day 7 and 14 was significantly reduced in IQGAP1^−/−^ mice as compared to wild type (WT) mice (left, Figure 1A). Postischemic blood flow recovery in IQGAP1^−/−^ mice gradually caught up to the level of WT mice at day 28, but it was still lower than that in WT mice. In addition, IQGAP1^−/−^ mice increased tissue damage, characterized by toe edema, degeneration on the nail beds and some degree of necrosis measured using a five-point scale (right, Figure 1A). Immunohistochemical analysis revealed that the numbers of CD31-positive capillary-like ECs as well as α-smooth muscle actin-positive arterioles in ischemic tissues at day 7 were significantly decreased in IQGAP1^−/−^ mice (Figure 1B). Histological analysis showed that increase in collateral lumen and wall area in semimembnosus muscle in WT mice [32] were significantly reduced in IQGAP1^−/−^ mice. On day 28, regeneration was widely observed in tibialis anterior muscles in WT mice, which was inhibited in IQGAP1^−/−^ mice that had more necrotic myofibers and large area of fibrosis (Supplemental Figure S1). Thus, IQGAP1 is involved in post-ischemic flow recovery by regulating angiogenesis and arteriogenesis as well as tissue repair and protection from necrosis.

### IQGAP1 Expression is increased in Ischemic Muscles after Hindlimb Ischemia

**Figure 2:**
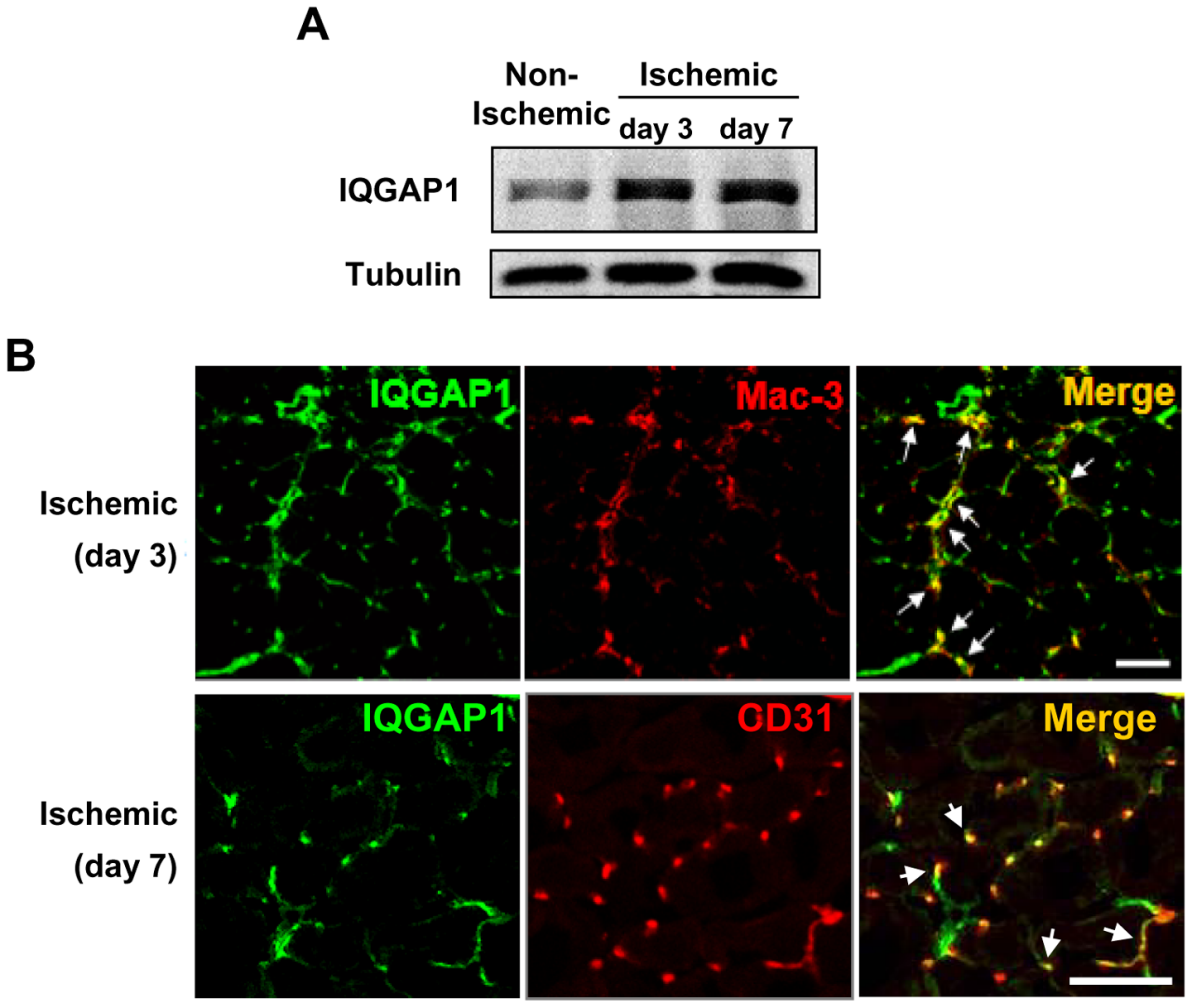
IQGAP1 expression is increased at macrophages and endothelial cells in ischemic hindlimbs. **A**, Representative Western blots for IQGAP1 or α-tubulin in adductor muscle at day 0, 3, and 7 after hindlimb ischemia. **B**, Representative staining for IQGAP1 (green) or Mac-3, macrophage marker (red) or their colocalization (merge) on day 3, and IQGAP1 (green) or CD31, EC marker (red) or their colocalization (merge) on day 7 in ischemic adductor muscles. Arrows represent colocalization with IQGAP1/Mac-3 or IQGAP1/CD31. Bars represent 100 µm.

We next examined if the levels of endogenous IQGAP1 are regulated after hindlimb ischemia *in vivo*. Western analysis showed that IQGAP1 protein expression was increased in ischemic muscles at days 3 and 7 after hindlimb ischemia (Figure 2A). Immunofluorescence analysis revealed that IQGAP1 protein expression was highly expressed in Mac-3^+^ macrophages on day 3 and CD31-positive ECs on day 7 in ischemic tissues (Figure 2B). These results suggest that tissue ischemia increases IQGAP1-positive inflammatory cells and ECs, which may contribute to neovascularization.

### IQGAP1 is involved in Macrophages Recruitment without affecting Mobilization of Monocytes or Angiogenic Cells after Ischemic Injury

**Figure 3:**
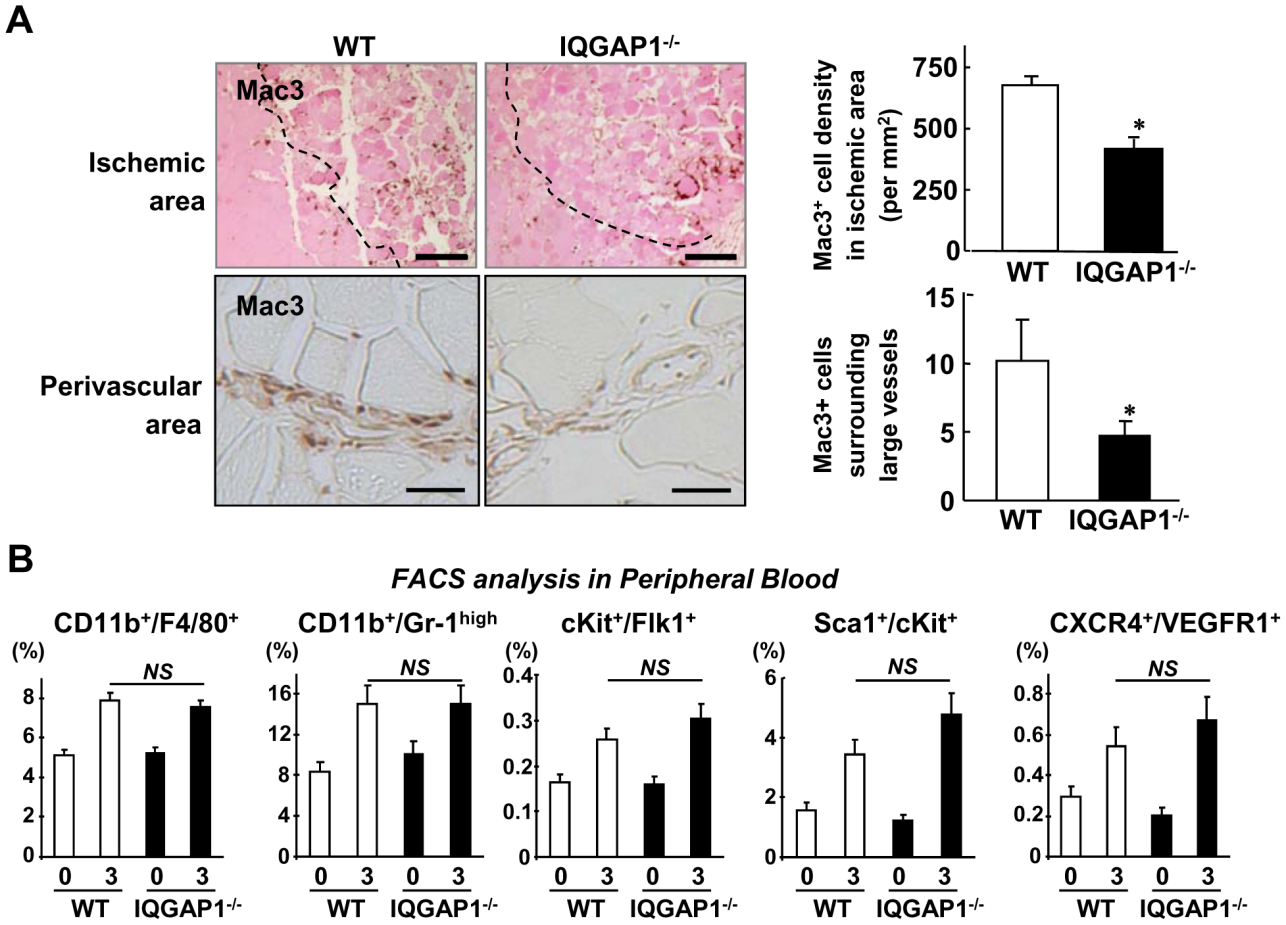
Macrophage infiltration into ischemic muscle, but not its mobilization into circulation, is impaired in IQGAP1^−/−^ mice. A, Immunostaining of Mac3 (monocyte/macrophage marker) in ischemic muscles from WT or IQGAP1^−/−^ mice at day 3. Upper, Mac3 positive cells in ischemic area lacking the original integrity of myofibers on eosin staining (upper left). The border of ischemic area is shown by dotted lines. Bars represent 100 µm. The density of Mac3 positive cells localized in the interstitium were determined (upper right, n = 3, *p<0.05). Lower, Mac3 positive cells in perivascular area of vessels in semimembranosus muscles (lower left). Bars represent 20 µm. The number of Mac3 positive cells distributed in the adventitia and the connective tissue surrounding the large vessels (>50 µm of diameter) were counted (lower right). B, Peripheral blood was obtained at day 0 (no ischemia surgery) and day 3. Total leukocytes were stained by fluorescence-conjugated various antibodies as indicated and analyzed by flow cytometory (n = 3 or 4 in each group). The data is expressed as the percentage of cells in total circulating leukocytes. Data shown are mean+/−SEM.

**Figure 4:**
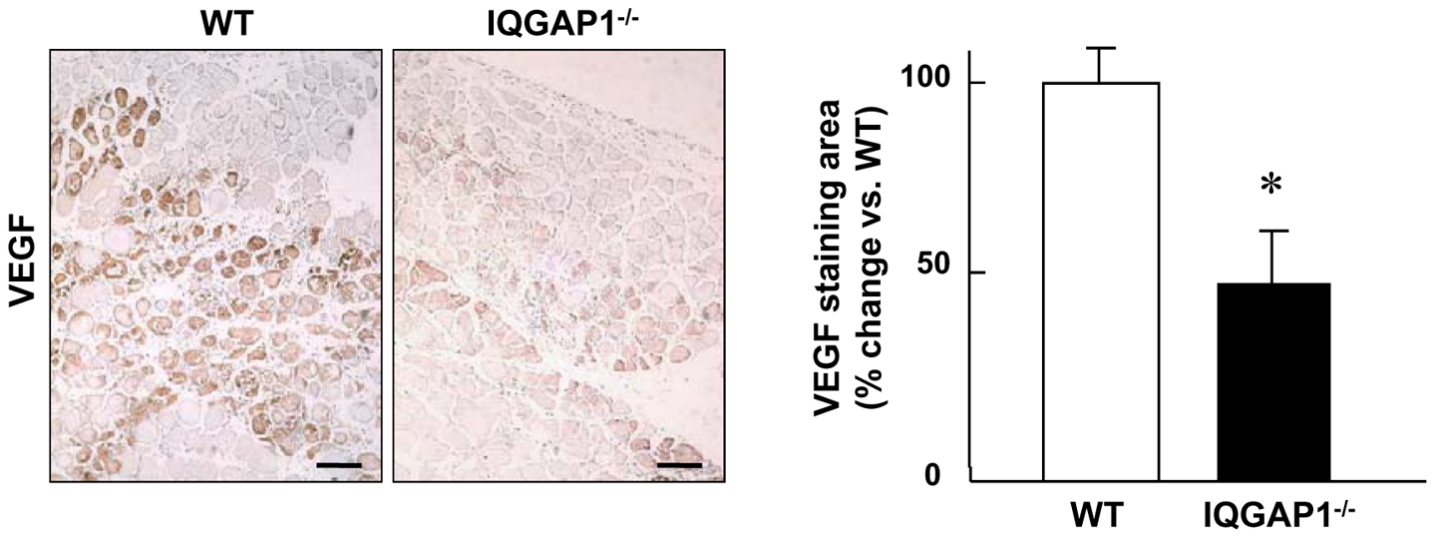
IQGAP1^−/−^ mice show reduced VEGF expression in ischemic muscle. Immunohistochemical staining of VEGF in ischemic adductor muscles at day 3. Brown staining shows VEGF positive cells and counter staining by hematoxylin was performed.

To determine the functional significance of upregulation of IQGAP1 in ischemic tissues, we next examined the role of IQGAP1 in inflammatory cell recruitment into ischemic sites. Figure 3A shows that Mac3^+^ cell recruitment to the ischemic area in the lower limbs (upper) and perivascular area in upper limbs (lower), which contribute to angiogenesis and arteriogenesis/collateral expansion [33], were significantly reduced in IQGAP1^−/−^ mice. Furthermore, we examined M1 and M2 phenotype of macrophages in ischemic muscles and found that the number of both pro-inflammatory macrophages/monocytes and anti-inflammatory macrophages was reduced in IQGAP1^−/−^ mice (Supplemental Figure S2A). In addition, expression of both pro-inflammatory cytokine (TNFα) and anti-inflammatory cytokine (IL-10) in ischemic tissues was decreased in IQGAP1^−/−^ mice, which was associated with reduced macrophage number, as measured by colony-stimulating factor receptor (c-fms) expression (Supplemental Figure S2B). These results suggest that IQGAP1 regulates macrophages infiltration without affecting their polarization capacity. Moreover, the levels of circulating monocytes (CD11b^+^/F4/80^+^ and CD11b^+^/Gr-1^high^) as well as endothelial progenitor cells (cKit^+^/Flk^+^), Sca1^+^/cKit^+^ progenitors or CXCR4^+^VEGFR1^+^ cells in peripheral blood at baseline or after ischemia were not different between WT and IQGAP1^−/−^ mice, as measured by FACS analysis (Figure 3B). We also found that VEGF expression in ischemic tissue was significantly decreased in IQGAP1^−/−^ mice (Figure 4).

### IQGAP1 is involved in ROS production after Hindlimb Ischemia

**Figure 5:**
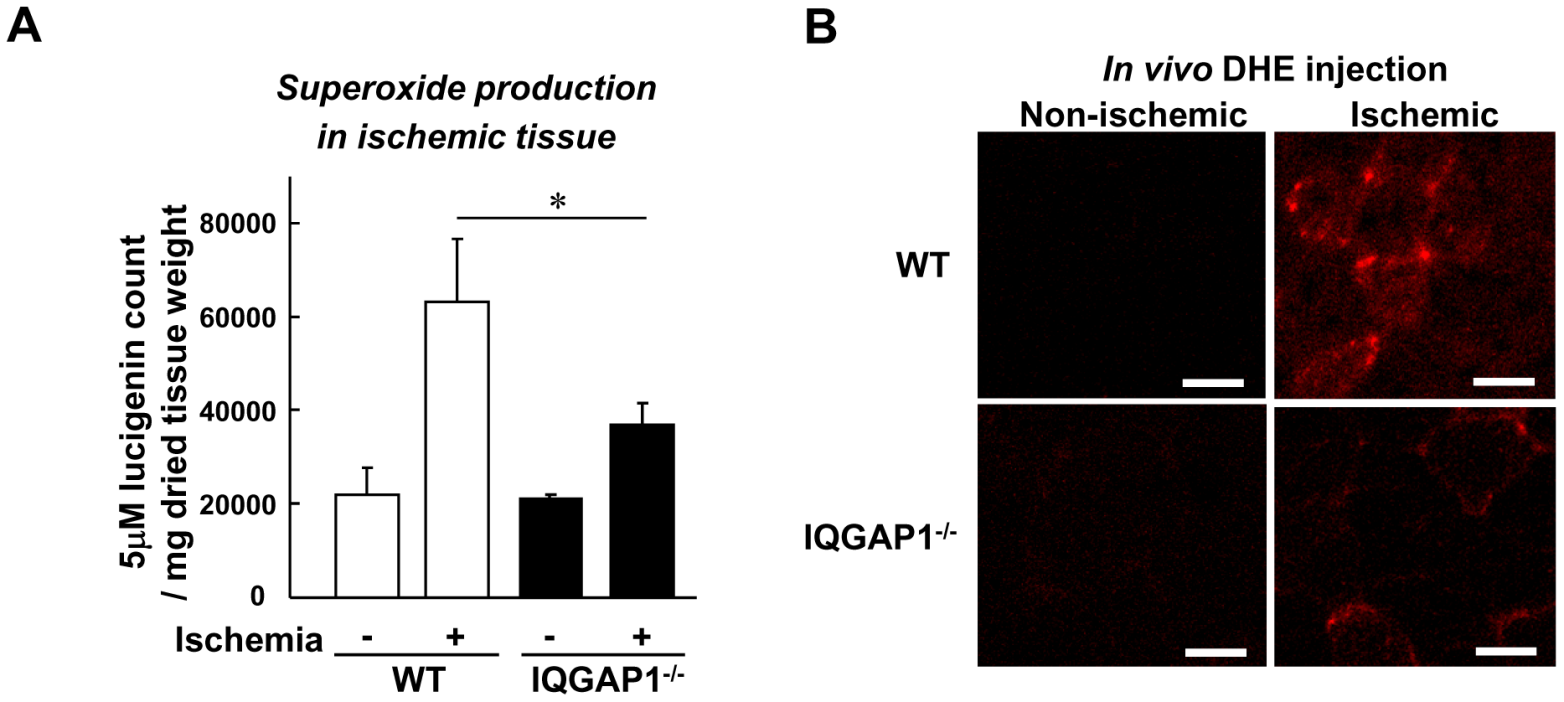
ROS production in ischemic muscles is impaired in IQGAP1^−/−^ mice. **A**, Superoxide (O_2_^−^) production was measured in ischemic muscles at day 0 (without surgery) and 7 days after hindlimb ischemia, using lucigenin chemiluminescence. The values were normalized by tissue dry weight (n = 4, *p<0.05). **B**, O_2_^−^ detection probe, dihydroethidium (DHE) was injected into mice at 30 minutes before sacrifice on day 7 after hindlimb ischemia, and tissues were harvested following perfusion fixation. DHE-positive signals (arrows) in non-ischemic and ischemic muscles of frozen sections (20 µm thickness) were observed with confocal microscope. Bars represent 20 µm.

Since IQGAP1 is expressed in both macrophages and capillary-like ECs (Figure 2), which are major sources of ROS in ischemic tissue [11], we next examined the role of IQGAP1 in ROS production induced by hindlimb ischemia. Figure 5A shows that ischemia-induced increase in O_2_^−^ production in ischemic tissue at day 7, as measured by lucigenin chemiluminescence assay, was significantly inhibited in IQGAP1^−/−^ mice. To confirm further this result, we also injected the O_2_^−^ specific dye, dihydroethidium (DHE), into the mice [28] and found that ischemia-induced increase in DHE staining *in situ* was markedly reduced in IQGAP1^−/−^ mice (Figure 5B).

### IQGAP1 in both BM-derived and Tissue Resident Cells is required for Ischemia-induced Neovascularization

**Figure 6:**
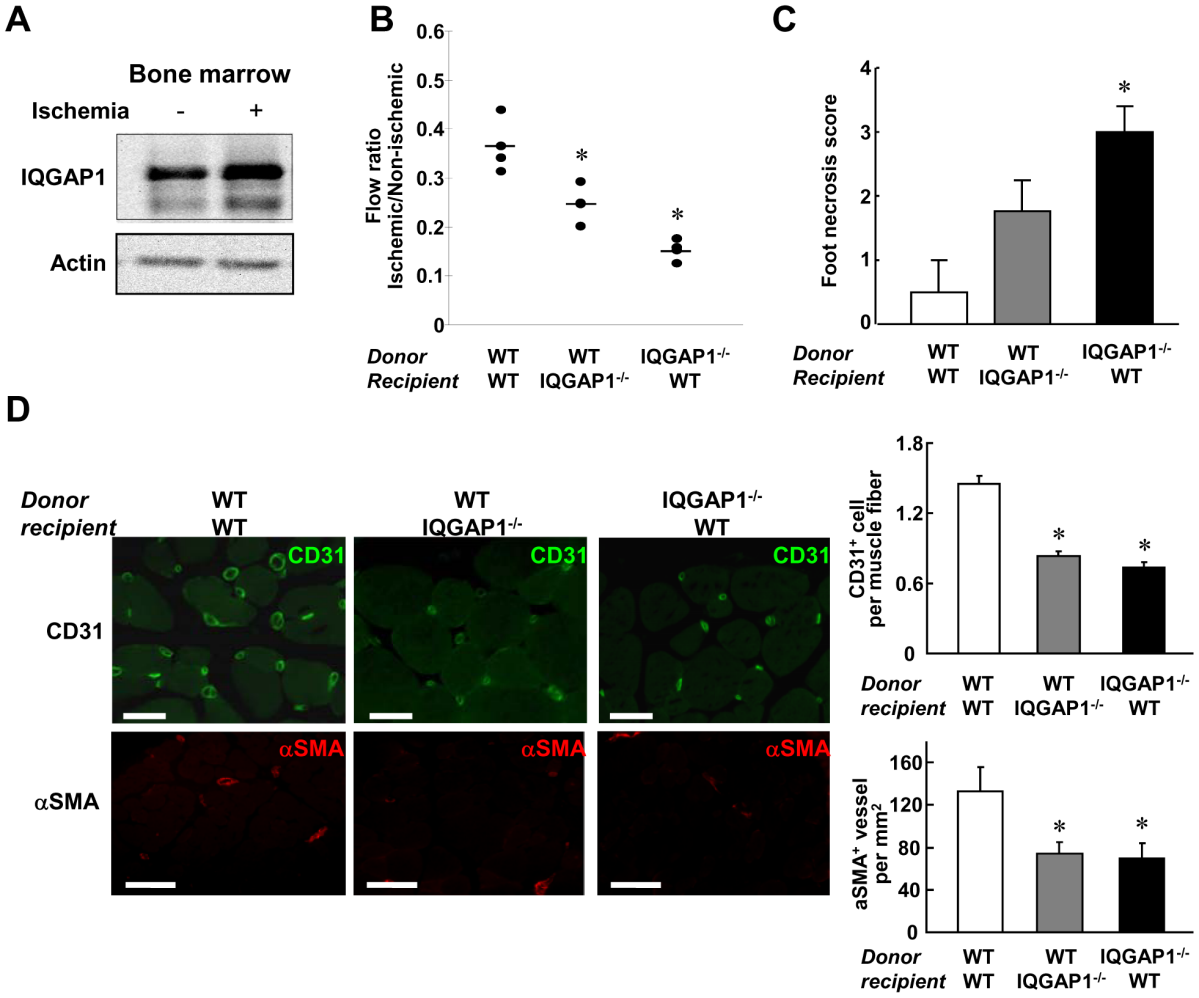
IQGAP1^−/−^ mice show reduced macrophage recruitment and ROS production in thioglycollate-elicited peritonitis model. Macrophage recruitment was induced by intraperitoneal (i.p.) injection of 4% thioglycollate (+) or PBS (−) into WT and IQGAP1^−/−^ mice. **A**, Number of macrophages recruited to the peritoneal cavity at 4 days after thioglycollate injection (n = 4, **p<0.01). **B**, Number of leukocytes in peripheral blood collected from the same animals as shown in A. **C**, O_2_^−^ production in recruited leukocytes at 4 days after injection, as measured by lucigenin chemiluminescence assay (n = 4 total, *p<0.05). The values were normalized by the number of peritoneal macrophages.

Since we found that IQGAP1 is highly expressed in both ECs and monocytes/macrophages in ischemic tissue, we next examined the relative role of IQGAP1 in BM-derived cells (BMCs) and tissue resident cells in post-ischemic neovascularization. Western analysis confirmed that IQGAP1 is abundantly expressed in BMCs, which is further upregulated by contra lateral hindlimb ischemia (Figure 6A). We thus performed BM transplantation (BMT) between WT and IQGAP1^−/−^ mice. Reconstitution of WT BM into lethally irradiated IQGAP1^−/−^ mice or IQGAP1^−/−^ BM into irradiated WT mice showed reduction in blood flow recovery (Figure 6B), increased tissue necrosis score (Figure 6C), as well as decreased number of CD31^+^ capillary like ECs and αSMA^+^ arterioles (Figure 6D) as compared to control group (WT mice reconstituted with WT-BM). These results suggest that IQGAP1 expressed in both BM-derived circulating cells and tissue resident cells including ECs contributes to ischemia-induced neovascularization.

### IQGAP1 is involved in Macrophage Recruitment and ROS production in Thioglycollate-elicited Peritonitis Model

**Figure 7:**
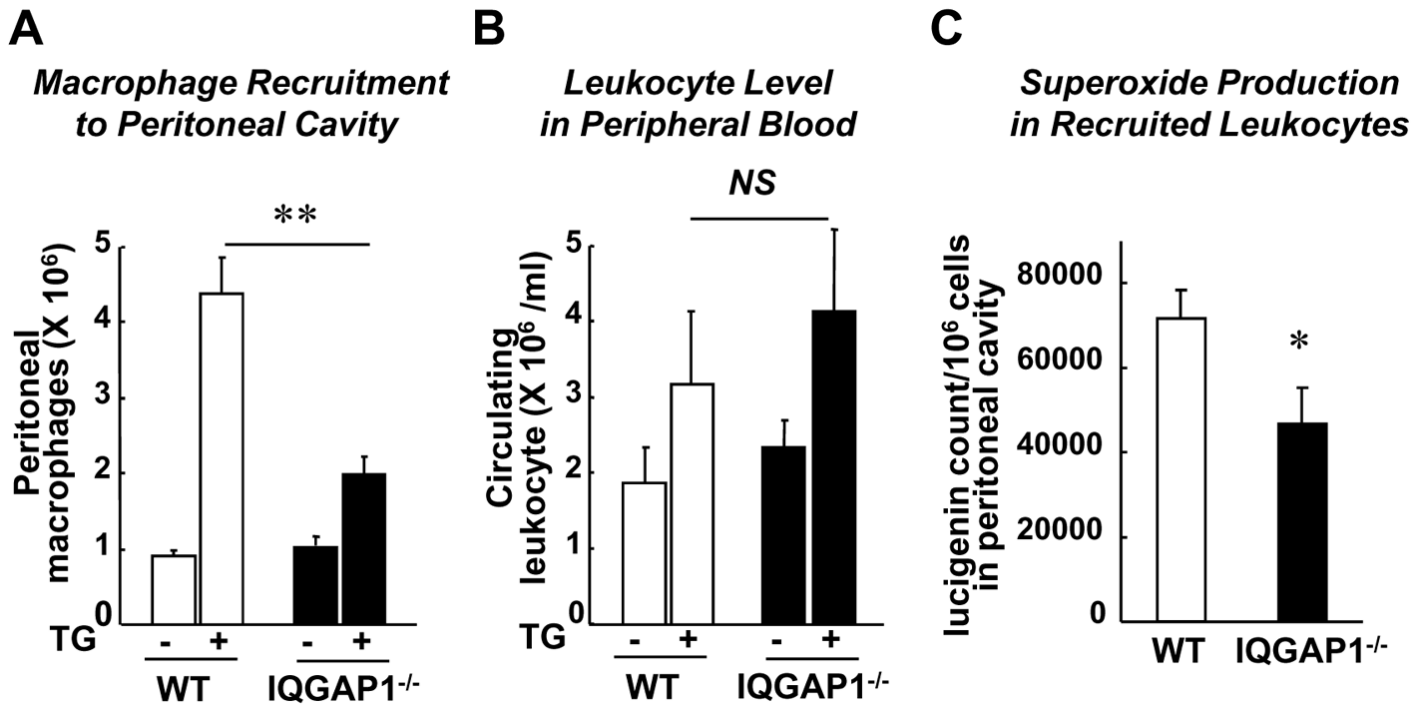
IQGAP1 in both bone marrow-derived and tissue resident cells is required for ischemia-induced neovascularization. **A**, Western analysis for IQGAP1 protein expression in whole bone marrow lysate (40 µg protein) obtained from femurs of healthy leg in mice subjected to contralateral hindlimb ischemia (day 3). Actin was used as loading control. **B–D**, Bone marrow transplantation (BMT) was performed between WT and IQGAP1^−/−^ mice. After 6 weeks of irradiation, BMT mice were subjected to hindlimb ischemia and observed for 21 days. Blood flow measured at day 21 (n = 4, *p<0.05 vs. WT-WT)(**B**), necrosis score as Figure 1 (n = 4, *p<0.05 vs. WT-WT)(**C**), and representative images of ischemic muscles stained by anti-CD31- (green) or anti-αSMA- (red) antibodies (**D**, left). Bars show 50 µm. In **D** (right), the numbers of CD31 positive ECs per muscle fiber or αSMA positive vessels per mm^2^ in ischemic muscles were obtained (n = 4, *p<0.05 vs. WT-WT). Data shown are mean+/−SEM.

To address further the role of IQGAP1 in recruitment of inflammatory cells, we used a thioglycollate-elicited peritonitis model to induce non-bacterial inflammation [27]. Injection of thioglycollate increased the number of macrophages in the peritoneal cavity compared with PBS injection in WT mice, which was significantly inhibited in IQGAP1^−/−^ mice (Figure 7A). By contrast, either neutrophil recruitment to the peritoneal cavity (data not shown) or the number of leukocyte in peripheral blood before and after thioglycollate challenge (Figure 7B) was not changed in IQGAP1^−/−^ mice. We also found that O_2_^−^ levels in macrophages obtained from peritoneal cavity were significantly reduced in IQGAP1^−/−^ cells (Figure 7C). Subpopulation analysis for the monocytes/macrophages in the peritoneal cavity showed that there was no significant difference for the pro-inflammatory and anti-inflammatory phenotypes between WT and IQGAP1^−/−^ mice (Supplemental Figure S2C). Thus, IQGAP1 seems to be involved in macrophage recruitment and ROS production without affecting macrophage polarization.

### IQGAP1 is involved in Migration and Adhesion of BM-derived Macrophages

**Figure 8:**
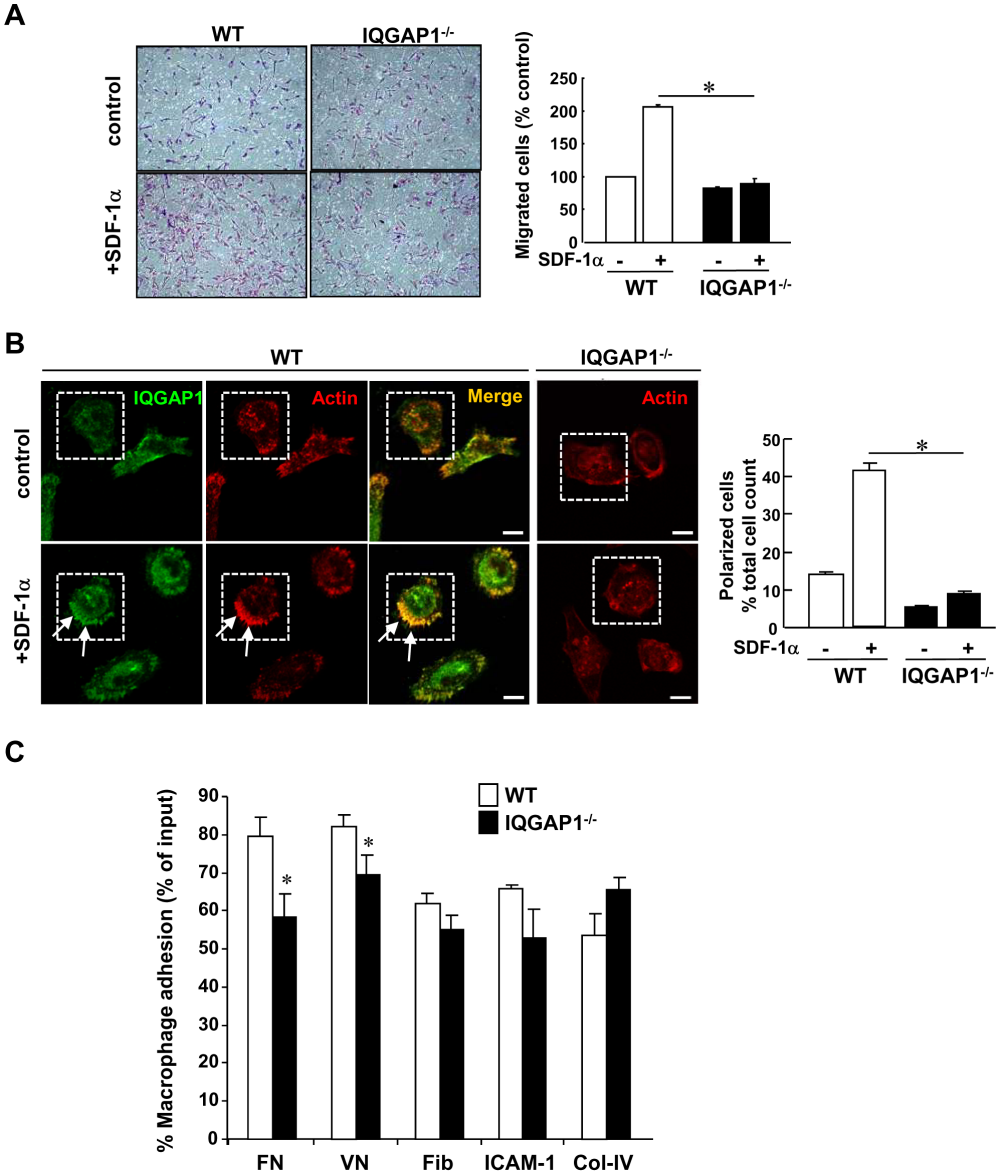
Migration capacity and actin polarization are impaired in IQGAP1^−/−^ BM-derived macrophages *in vitro*. **A**, WT and IQGAP1^−/−^ BM-derived macrophages (BMMs) were placed on upper Boyden chambers, and 100 ng/ml SDF-1α was placed in lower chambers for 4 hours. The number of migrated cells was counted and expressed as percent change vs. unstimulated WT cells (control) (n = 3, *p<0.05). **B**, Left, representative images of co-staining for anti-IQGAP1 (green) and Alexa Fluor 568 conjugated phalloidin (red) in WT-BMMs stimulated with SDF-1α (100 ng/mL, 2 min), as visualized by confocal microscopy. Arrows indicate colocalization of F-actin with IQGAP1 at the polarized plasma membrane in SDF-1α-stimulated BMMs. Phalloidin staining in IQGAP1^−/−^ BMMs is shown in the right panels. Right, quantitative analysis of polarized F-actin in WT- and IQGAP1^−/−^ BMMs, expressed as percentage of the total cell number at 16 different high power fields (×100)(*p<0.05). Bars show 20 µm. **C**, Adhesion capacity of bone marrow-derived macrophages (BMMs) to various extracellular matrixes. Cells were replated onto wells coated with fibronectin (FN), vitronectin (VN), fibrinogen (Fib), ICAM-1 or collagen IV (ColIV). After 30 minutes incubation, cells were washed by PBS gentry, fixed with 4% paraformaldehyde and stained with 0.05% crystal violet. Eluted dyes were measured by O.D. 590 nm. Data represents the means+/−SEM by duplicated samples and expressed as % cell adhesion vs. total input (*p<0.05).

To determine the mechanism by which IQGAP1 regulates macrophage recruitment, we examined the role of IQGAP1 in macrophage migration. BMCs isolated from mice were cultured for 7 days in the presence of macrophage colony stimulating factor 1 (MCSF1) to differentiate into monocytes/macrophage as described previously [29]. The differentiation capacity into macrophages was similar between WT- and IQGAP1^−/−^ BMCs, as measured by the percentage of CD11b and F4/80 expressions with flow cytometry analysis (Supplemental Figure S3A). However, the morphology of IQGAP1^−/−^ BM-derived macrophages (BMMs) attached on the culture dish or the glass cover slip were rounder compared to WT cells (Supplemental Figure S3B). Modified Boyden chamber assay showed that IQGAP1^−/−^ BMMs had impaired migration in response to chemokine, SDF-1α (Figure 8A), but not to VEGF (Supplemental Figure S4B). Of note, SDF-1α expression in ischemic tissues was not significantly different between WT and IQGAP1^−/−^ mice (data not shown), while VEGF expression was markedly reduced in IQGAP1^−/−^ mice (Figure 4). Thus, endogenous IQGAP1 is involved in SDF-1/CXCR4-mediated migration of BMMs, thereby promoting infiltration of macrophages into the ischemic tissues. Since actin polymerization is an important mechanism for migration, and IQGAP1 is known to directly bind to F-actin, we next examined the role of IQGAP1 in BMMs for this response. Immunofluorescence analysis showed that IQGAP1 was colocalized with F-actin at the plasma membrane in SDF-1α-stimulated BMMs (Figure 8B). SDF-1α-induced actin polarization was markedly inhibited in IQGAP1^−/−^ macrophages (Figure 8B). Thus, IQGAP1 is involved in chemotaxis of BM macrophages at least in part by regulating actin reorganization. Further, we assessed adhesion capacity of BMMs. We found that IQGAP1^−/−^ BMMs had a lower adhesion capacity to fibronectin and vitronectin as compared to WT BMMs (Figure 8C).

## Discussion

The present study uncovers a novel role of IQGAP1 in post-ischemic neovascularization and tissue repair by regulating not only angiogenesis but also macrophage infiltration to ischemic tissues. Here we show that: 1) Hindlimb ischemia increases IQGAP1 expression in infiltrating macrophages and ECs in ischemic tissues; 2) Mice lacking IQGAP1 show decreased blood flow recovery, capillary density and α-SMA-positive arterioles, and increased damage in ischemic tissue; 3) Macrophage recruitment and ROS production in ischemic tissue, but not the number of circulating monocytes, are significantly inhibited in IQGAP1^−/−^ mice; 4) BM reconstitution experiments show that IQGAP1 in both BM-derived and tissue resident cells contribute to capillary formation and α-SMA positive pericyte recruitment in ischemic tissue; 5) Mechanistically, IQGAP1^−/−^ BM-derived macrophages show an inhibition of migration and adhesion capacity.

Post-ischemic neovascularization is a complex process coordinated by angiogenic ECs and recruited inflammatory cells. We and others have previously demonstrated that IQGAP1 directly binds to VEGFR2 or cSrc and B-Raf, and knockdown of endogenous IQGAP1 with siRNA inhibits VEGF-induced angiogenic responses in cultured ECs [18], [19], [20]. We also reported that IQGAP1 expression is upregulated in regenerating ECs in response to vascular [18] or ischemic injury [21]. However, a role of endogenous IQGAP1 in ischemia-induced neovascularization was virtually unexplored. Here we provide compelling evidence that ischemia-induced increases in CD31^+^ capillary-like ECs (angiogenesis) and α-SMA^+^ arterioles (pericyte recruitment) are significantly reduced in IQGAP1^−/−^ mice, thereby inhibiting blood flow recovery at days 7 and 14 after ischemia. Consistent with our results, Meyer et al. recently reported that depletion of IQGAP1 with siRNA suppresses VEGF-induced angiogenesis in an *in vivo* model of chicken chorioallantoic membrane [19]. In the current study, postischemic blood flow recovery in IQGAP1−/− mice gradually catch up to that of WT mice at day 28 but not to the level seen in WT mice. Together with histological analysis, these results suggest that IQGAP1 plays an important role in post-ischemic neovascularization *in vivo* by regulating arteriogenesis and angiogenesis.

The present study also demonstrates that IQGAP1 expression is increased in ischemic tissues, which may be due to increased, infiltrated macrophages and newly formed ECs, which express IQGAP1, at day 3 and 7, respectively. Functionally, IQGAP1−/− mice show impaired recruitment of monocytes/macrophages into ischemic tissue and perivascular areas in the upper limbs, which is associated with lower expression of VEGF. It has been shown that macrophage-derived VEGF contributes to angiogenesis and arteriogenesis in ischemic tissues [1], [2], [3], [4] and that the recruitment of macrophages participate in arteriogenesis, blood flow recovery, and angiogenesis [34]. Thus, IQGAP1-dependent inflammatory cells recruitment maybe required for post-ischemic neovascularization. The role for IQGAP1 in BM-derived macrophages and ECs in the hindlimb ischemia model is demonstrated by BMT experiments. Transfer of WT-BM to recipient IQGAP1^−/−^ mice or IQGAP1^−/−^ BM to WT recipients lowers blood flow recovery, capillary density and α-SMA^+^ arterioles in ischemic tissues than WT mice transplanted with WT-BM. These results suggest that IQGAP1 expressed in both BM-derived cells and tissue resident cells, including ECs, plays an important role in reparative neovascularization in response to tissue ischemia. We cannot exclude the possible involvement of IQGAP1 in tissue resident macrophages in this study.

Infiltrated macrophages are also critical to tissue healing and regeneration after ischemic injury [3], [5], [6], [7]. The present study demonstrates that regeneration of skeletal muscle is impaired in association with an increase in necrosis and fibrosis in IQGAP1^−/−^ mice at later phase after hindlimb ischemia, suggesting that IQGAP1 plays an important role in macrophage function. This is consistent with a previous study showing reduced phagocytotic activity of IQGAP1-depleted RAW macrophages *in vitro* [17]. Thus, IQGAP1-mediated inflammatory cell recruitment may contribute to angiogenesis and repair/regeneration of muscle, thereby promoting restoration of perfusion in the ischemic limbs. In this study, we found that the lack of IQGAP1 does not affect mobilization of BM-derived cells including monocytes and other angiogenic cells to peripheral blood after hindlimb ischemia. Similar results were obtained in a non-bacterial inflammation peritonitis model, where thioglycollate-elicited peritoneal macrophage infiltration to tissue is inhibited in IQGAP1^−/−^ mice, with no difference in the level of circulating leukocytes. Furthermore, M1 and M2 phenotype analysis of macrophages in ischemic tissues and peritoneal macrophages reveal that IQGAP1 is seemingly not involved in macrophage polarization. Thus, these results indicate that IQGAP1 is generally involved in macrophage recruitment during inflammation without affecting their mobilization from BM or polarization, which may contribute to neovascularization and tissue repair in response to injury.

The present study also shows that ROS production in ischemic muscle, as well as “activated and tissue infiltrated” macrophage obtained by peritonitis inflammation model, is inhibited in IQGAP1^−/−^ mice. We previously reported that ROS produced by NADPH oxidase expressed in inflammatory cells and ECs play a critical role in ischemia-induced neovascularization [11]. Moreover, IQGAP1 is shown to be involved in VEGF [18]- and hyperoxia [35]-induced ROS production in cultured ECs. Thus, the reduction of ROS at sites of inflammation and neovascularization in IQGAP1^−/−^ mice may be at least due to a decrease in the number of infiltrated inflammatory cells and angiogenic ECs as well as their ROS producing capacity. A recent study reveals that a tissue gradient of ROS production represents a critical signal for recruiting leukocytes to injury sites *in vivo* [36]. Therefore, it is tempting to speculate that an IQGAP1-dependent increase in ROS signal from inflammatory cells and angiogenic ECs at ischemic sites may further facilitate the recruitment of monocytes/macrophages, thereby promoting revascularization and tissue repair/muscle regeneration.

To determine the mechanism by which IQGAP1 is involved in inflammatory cell recruitment, we examined the role of IQGAP1 in macrophage migration *in vitro*. IQGAP1 has been shown to cross-link actin filaments, and plays a key role in cell motility in various systems, including fibroblasts, cancer cells and ECs [15], [16], [18], [20], [22], [37], [38]. However, a role of IQGAP1 in the function of intact macrophages has not been investigated. Using cultured BM-derived macrophages (BMMs) derived from WT and IQGAP1^−/−^ mice, the present study demonstrates that IQGAP1 is involved in macrophage migration in response to SDF-1α, but not to VEGF, thereby promoting macrophage infiltration to ischemic tissues. Given that macrophages mainly expresses VEGF receptor type 1 (VEGFR1) instead of VEGFR2, which is expressed in ECs, it is likely that IQGAP1 is coupled to VEGFR2 [18], [19], but not to VEGFR1. In macrophages, IQGAP1 seems to be important mediator for SDF-1/CXCR4 signaling involved in cell migration. Investigating this point in detail is the subject of future studies. In ischemic tissues, expression of VEGF, but not SDF-1α, is reduced in IQGAP1^−/−^ mice, which may contribute to impaired angiogenesis.

Mechanistically, IQGAP1 colocalizes with F-actin at the plasma membrane during active migration, and SDF-1α-induced actin polarization is inhibited in IQGAP1^−/−^ BMMs. In line with these findings, we previously reported that IQGAP1 translocates to the leading edge where it colocalizes with VEGFR2 and NADPH oxidase in ECs, thereby promoting directional EC migration [18], [20]. In RAW macrophage, IQGAP1 directly binds to Dia1 at the actin-rich phagocytic cup, which is required for migration and phagocytosis [17]. IQGAP1 also directly binds to the active form of Rac, Arp2/3 and N-WASP, thereby regulating actin reorganization in active migrating cells [13], [15], [16]. In addition, we found that the lack of IQGAP1 impairs fibronectin- or vitronectin-mediated macrophage adhesion. IQGAP1 association with β1 integrin has been reported previously [39]. Our results suggest that IQGAP1 may couple to other integrins to regulate macrophage adhesion. This point requires further investigation. Taken together, the present study indicates that IQGAP1 is involved in macrophage migration and adhesion, thereby promoting inflammatory cell infiltration into ischemic tissues, which may contribute to post-ischemic revascularization and tissue repair. It is possible that IQGAP1 expressed in inflammatory cells and/or ECs may regulate cross-talk between these cells, as reported for the role of neutrophil NADPH oxidase in activation of redox signaling in ECs [40] or endothelial IQGAP1 in lymphocyte transendothelial migration [41].

In summary, the present study provides compelling evidence that IQGAP1 plays a critical role in post-ischemic neovascularization and tissue repair by regulating inflammatory cell infiltration, EC-mediated angiogenesis and ROS production in ischemic tissues. We also found that IQGAP1-mediated recruitment of monocytes/macrophages into ischemic tissues is, in part, due to promoting their migration and adhesion capacity. These findings provide novel insight into IQGAP1 as a potential therapeutic target for inflammation- and angiogenesis-dependent ischemic diseases including peripheral vascular disease, arteriosclerosis, and wound healing, as well as tumor progression.

## Supporting Information

Figure S1 Impaired regeneration as well as increased necrosis and fibrosis in ischemic muscles in IQGAP1^-/-^ mice. A, Sections of muscles in lower limb were stained with hematoxyline and eosin (H/E). Regenerated myofibers which have centered nuclei (arrows) and ghost muscle cells devoid of nucleus (asterisk) are indicated. Adipocytes infiltration is indicated by arrow heads. The area of regeneration and necrosis including necrotic myofibers and infiltrated adipocytes are measured in tibialis anterior (TA) muscles which consistently show inflammation and necrotic damage in WT mice. B, serial sections of A were stained by Masson Trichrome. Blue indicates fibrosis area in TA muscles (n = 3-4, *p<0.05). Data shown are mean+/-SEM.
(0.11 MB PDF)

Figure S2 A, Upper panel: representative dot plots of F4/80 and Ly-6C expression in CD11b^+^/Ly-6G^-^ population WT and IQGAP1^-/-^ mice in ischemic tibialis anterior muscles at day 4. Cell suspension was obtained from ischemic muscles using collagenase treatment and labeled with specific antibodies against CD45, CD11b, Ly-6C, Ly-6G and F4/80. Lower panel: total infiltrated cell numbers for pro-inflammatory M1 macrophages (CD11b^+^Ly6G^-^Ly6C^+^F4/80^+^; Ly-6C^+^ MP) and monocytes (CD11b^+^Ly6G^-^Ly6C^+^F4/80^-^; Ly-6C^+^ MO) as well as anti-inflammatory M2 macrophages (CD11b^+^Ly6G^-^Ly6C^-^F4/80^+^; Ly-6C^-^ MP) and monocytes (CD11b^+^Ly6G^-^Ly6C^-^F4/80^-^; Ly-6C^-^ MO) were calculated based on tissue weight and total cell numbers. Means+/-SEM from 2 different mice are shown. B, Pro-inflammatory M1 cytokines (TNFα and IL-6) and anti-inflammatory M2 cytokine (IL-10) expressions in ischemic muscles were measured by PCR analysis. Total RNA was extracted from muscle tissues and the data were normalized by GAPDH gene expression. Colony-stimulating factor receptor (c-fms) expression indicates macrophages infiltration. The relative gene expressions vs. WT are shown (n = 4 in each group, *p<0.05). C, Total cells collected from peritoneal cavities at 3 days after thioglycollate challenge were analyzed for CD11b, Ly-6G, Ly-6C and F4/80 expression by flow cytometry. The percentages of inflammatory (Ly-6C^+^) monocytes (MO) or macrophages (MP) and antiinflammatory (Ly-6C^-^) MO or MP were shown. Results represent the means+-SEM from 2 different mice in each group.
(0.08 MB PDF)

Figure S3 A, Expression of CD11b (Mac-1) and F4/80 on cultured bone marrow-derived macrophages (BMMs). BMMs were stained with anti-CD11b and anti-F4/80 antibodies or isotype control antibodies and subjected to flow cytometric analysis. The values in right upper and right lower quadrants shows representative percentage of CD11b and F4/80 double positive, and CD11b positive but F4/80 negative in the total cells. B, The representative pictures of BMMs from WT and IQGAP1^-/-^ mice at day 7 of primary cultured in the presence of 5% serum and 20 ng/ml of M-CSF (growing condition). The elongation ratio (ratio between longest and shortest cell axis) was measured. The result is expressed by mean ± SEM of ≧70 cells from each population over three separate experiments (p<0.01).
(0.05 MB PDF)

Figure S4 WT and IQGAP1^-/-^ BM-derived macrophages (BMMs) were placed on upper Boyden chambers, and 100 ng/ml SDF-1α or 50 ng/ml VEGF was placed in lower chambers for 6 hours. The number of migrated cells was counted in high power field (X20 objective). Data were obtained at least 3 independent cultures in each group from duplicated samples. N.S. represents non-significant.
(0.10 MB PDF)
