# Supplementary figures and images for: IQGAP1 Is Involved in Post-Ischemic Neovascularization by Regulating Angiogenesis and Macrophage Infiltration
Source: PLoS One. 2010 Oct 15;5(10):e13440. doi: 10.1371/journal.pone.0013440 (PMC2955540; doi:10.1371/journal.pone.0013440)

**Figure S1**

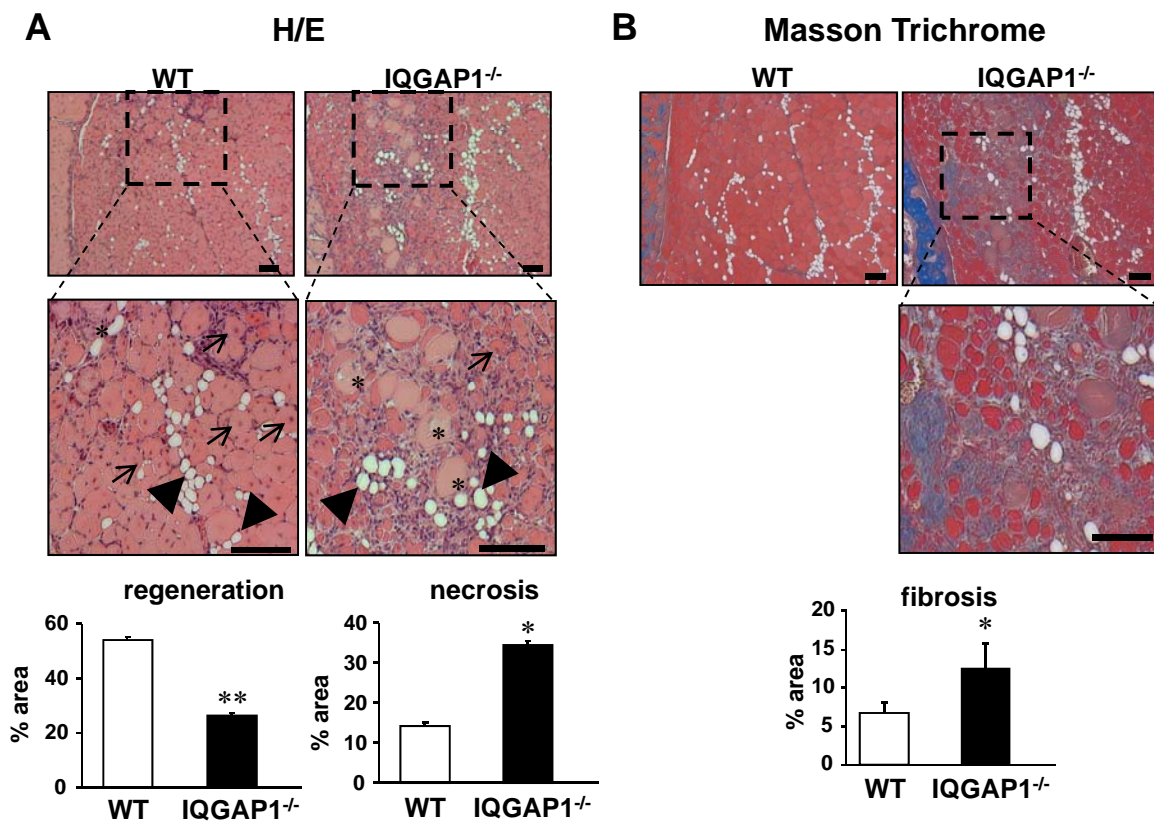

Supplement: Figure S1 — Impaired regeneration as well as increased necrosis and fibrosis in ischemic muscles in IQGAP1-/- mice. A, Sections of muscles in lower limb were stained with hematoxyline and eosin (H/E). Regenerated myofibers which have centered nuclei (arrows) and ghost muscle cells devoid of nucleus (asterisk) are indicated. Adipocytes infiltration is indicated by arrow heads. The area of regeneration and necrosis including necrotic myofibers and infiltrated adipocytes are measured in tibialis anterior (TA) muscles which consistently show inflammation and necrotic damage in WT mice. B, serial sections of A were stained by Masson Trichrome. Blue indicates fibrosis area in TA muscles (n = 3-4, *p<0.05). Data shown are mean+/-SEM. (0.11 MB PDF) [file pone.0013440.s001.pdf]

**Figure S2**

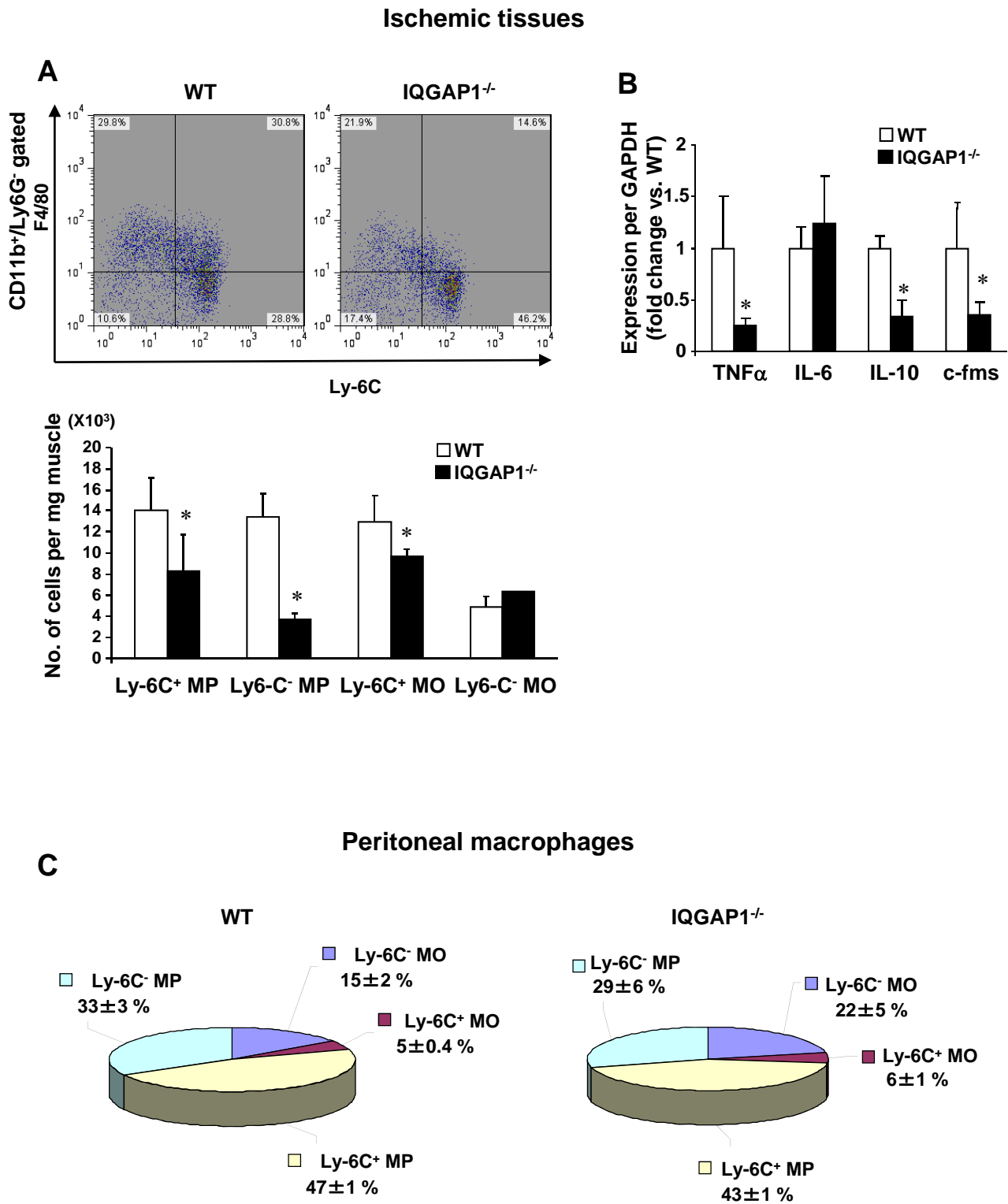

Supplement: Figure S2 — A, Upper panel: representative dot plots of F4/80 and Ly-6C expression in CD11b+/Ly-6G- population WT and IQGAP1-/- mice in ischemic tibialis anterior muscles at day 4. Cell suspension was obtained from ischemic muscles using collagenase treatment and labeled with specific antibodies against CD45, CD11b, Ly-6C, Ly-6G and F4/80. Lower panel: total infiltrated cell numbers for pro-inflammatory M1 macrophages (CD11b+Ly6G-Ly6C+F4/80+; Ly-6C+ MP) and monocytes (CD11b+Ly6G-Ly6C+F4/80-; Ly-6C+ MO) as well as anti-inflammatory M2 macrophages (CD11b+Ly6G-Ly6C-F4/80+; Ly-6C- MP) and monocytes (CD11b+Ly6G-Ly6C-F4/80-; Ly-6C- MO) were calculated based on tissue weight and total cell numbers. Means+/-SEM from 2 different mice are shown. B, Pro-inflammatory M1 cytokines (TNFα and IL-6) and anti-inflammatory M2 cytokine (IL-10) expressions in ischemic muscles were measured by PCR analysis. Total RNA was extracted from muscle tissues and the data were normalized by GAPDH gene expression. Colony-stimulating factor receptor (c-fms) expression indicates macrophages infiltration. The relative gene expressions vs. WT are shown (n = 4 in each group, *p<0.05). C, Total cells collected from peritoneal cavities at 3 days after thioglycollate challenge were analyzed for CD11b, Ly-6G, Ly-6C and F4/80 expression by flow cytometry. The percentages of inflammatory (Ly-6C+) monocytes (MO) or macrophages (MP) and antiinflammatory (Ly-6C-) MO or MP were shown. Results represent the means+-SEM from 2 different mice in each group. (0.08 MB PDF) [file pone.0013440.s002.pdf]

**Figure S3**

**A**

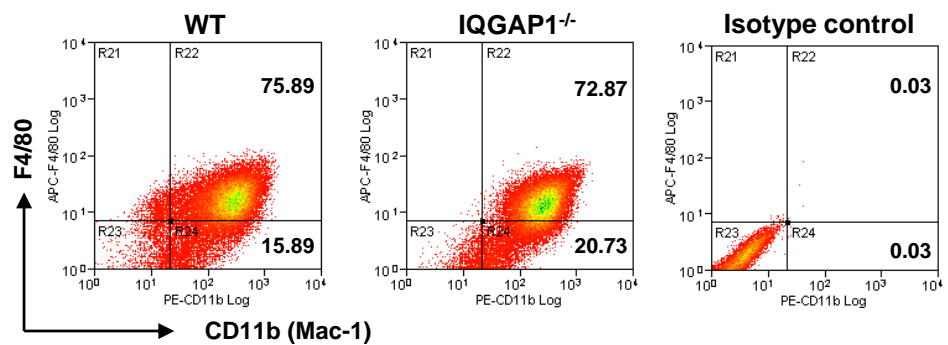

**B**

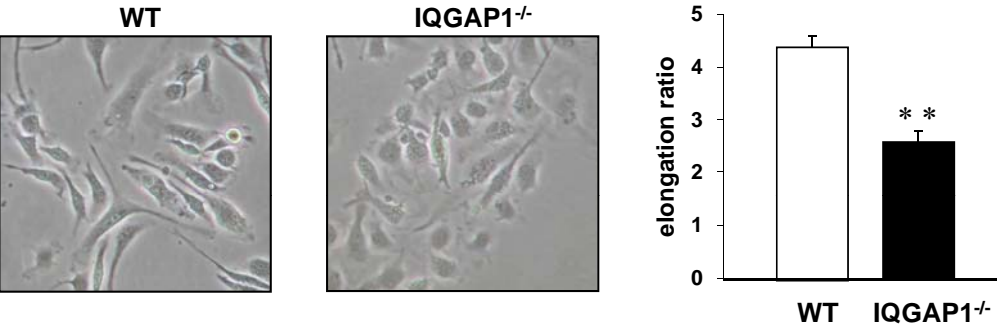

Supplement: Figure S3 — A, Expression of CD11b (Mac-1) and F4/80 on cultured bone marrow-derived macrophages (BMMs). BMMs were stained with anti-CD11b and anti-F4/80 antibodies or isotype control antibodies and subjected to flow cytometric analysis. The values in right upper and right lower quadrants shows representative percentage of CD11b and F4/80 double positive, and CD11b positive but F4/80 negative in the total cells. B, The representative pictures of BMMs from WT and IQGAP1-/- mice at day 7 of primary cultured in the presence of 5% serum and 20 ng/ml of M-CSF (growing condition). The elongation ratio (ratio between longest and shortest cell axis) was measured. The result is expressed by mean ± SEM of ≧70 cells from each population over three separate experiments (p<0.01). (0.05 MB PDF) [file pone.0013440.s003.pdf]

**Figure S4**

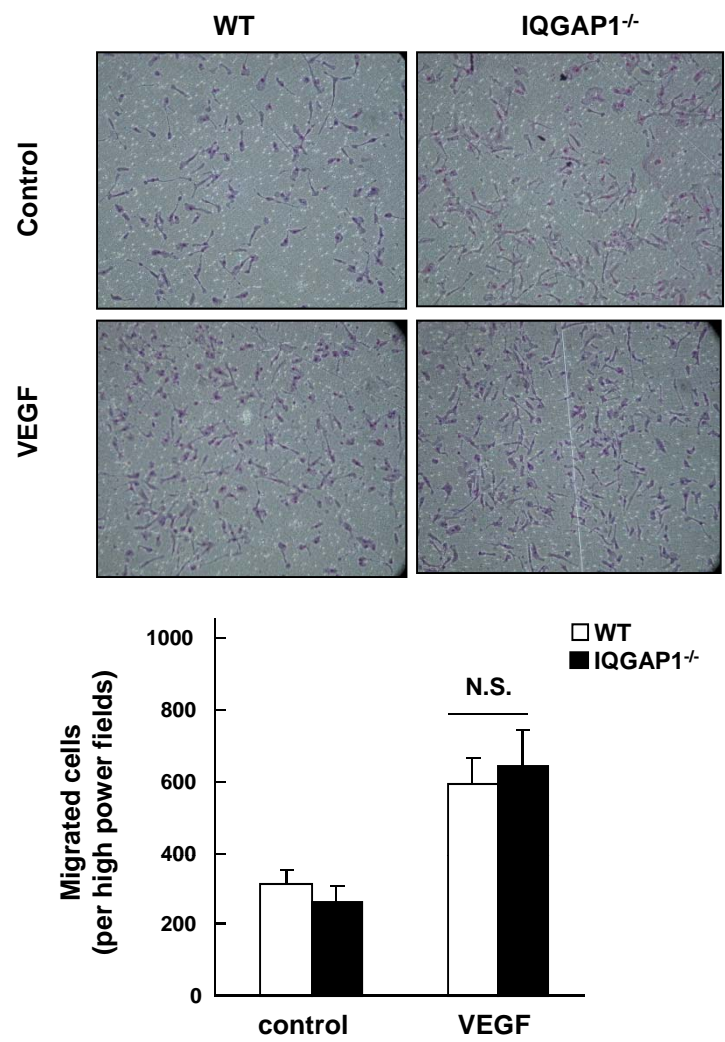

Supplement: Figure S4 — WT and IQGAP1-/- BM-derived macrophages (BMMs) were placed on upper Boyden chambers, and 100 ng/ml SDF-1α or 50 ng/ml VEGF was placed in lower chambers for 6 hours. The number of migrated cells was counted in high power field (X20 objective). Data were obtained at least 3 independent cultures in each group from duplicated samples. N.S. represents non-significant. (0.10 MB PDF) [file pone.0013440.s004.pdf]
